# Neural Control of Enhanced Filtering Demands in a Combined Flanker and Garner Conflict Task

**DOI:** 10.1371/journal.pone.0120582

**Published:** 2015-03-19

**Authors:** David Berron, Sascha Frühholz, Manfred Herrmann

**Affiliations:** 1 Department of Neuropsychology and Behavioral Neurobiology, Bremen University, Bremen, Germany; 2 Institute of Cognitive Neurology and Dementia Research, Otto-von-Guericke University, Magdeburg, Germany; 3 Center for Cognitive Sciences (ZKW), Bremen University, Bremen, Germany; 4 Swiss Center for Affective Sciences, University of Geneva, Geneva, Switzerland; Max Planck Institute for Human Cognitive and Brain Sciences, GERMANY

## Abstract

Several studies demonstrated that visual filtering mechanisms might underlie both conflict resolution of the Flanker conflict and the control of the Garner effect. However, it remains unclear whether the mechanisms involved in the processing of both effects depend on similar filter mechanisms, such that especially the Garner effect is able to modulate filtering needs in the Flanker conflict. In the present experiment twenty-four subjects participated in a combined Garner and Flanker task during two runs of functional magnetic resonance imaging (fMRI) recordings. Behavioral data showed a significant Flanker but no Garner effect. A run-wise analysis, however, revealed a Flanker effect in the Garner filtering condition in the first experimental run, while we found a Flanker effect in the Garner baseline condition in the second experimental run. The fMRI data revealed a fronto-parietal network involved in the processing of both types of effects. Flanker interference was associated with activity in the inferior frontal gyrus, the anterior cingulate cortex, the precuneus as well as the inferior (IPL) and superior parietal lobule (SPL). Garner interference was associated with activation in middle frontal and middle temporal gyrus, the lingual gyrus as well as the IPL and SPL. Interaction analyses between the Garner and the Flanker effect additionally revealed differences between the two experimental runs. In the first experimental run, activity specifically related to the interaction of effects was found in frontal and parietal regions, while in the second run we found activity in the hippocampus, the parahippocampal cortex and the basal ganglia. This shift in activity for the interaction effects might be associated with a task-related learning process to control filtering demands. Especially perceptual learning mechanisms might play a crucial role in the present Flanker and Garner task design and, therefore, increased performance in the second experimental run could be the reason for the lack of behavioral Garner interference on the level of the whole experiment.

## Introduction

A crucial ability of the human brain is to coordinate behavior in accordance with internal goals and the external context, a process often referred to as “cognitive control”. This term describes the cognitive and brain mechanisms in order to adjust the cognitive system to a currently prioritized task. Since the cognitive system has limited information processing resources, this adjustment is of particular importance for goal-intended behavior [1]. Cognitive control has been widely studied using cognitive conflict tasks such as the Eriksen flanker task [2], the Simon task [3], or the classical Stroop [4] and Stroop-like tasks [5]. Because cognitive conflicts appear at different levels of information processing, it is still under debate whether the control mechanisms recruited by different cognitive conflicts are drawing on the same cognitive resources [6]. Previous studies using cognitive control tasks demonstrated evidence for conflict and domain specific control mechanisms in the human brain [6,7].

The present study aimed at investigating the underlying brain mechanisms for conflict processing during different filtering demands using the Eriksen flanker task [2] and the Garner effect [8,9] in a combined presentation of both tasks. Both tasks are assumed to rely on the ability to filter out task-irrelevant distractors [10,11]. The Eriksen flanker task can be interpreted as a conflict that arises at the perceptual level (stimulus-based) [7,11,12] as well as at the stage of response preparation (response-based) [12,13]. Studies investigating the differential contributions of stimulus-based and response-based conflict resolution-mechanisms in the Flanker task demonstrated that both type of conflicts contribute to the decrease in performance. Whereas the main source of conflict might be a response-based conflict at the stage of response selection there seems to be also a conflict at an earlier stimulus processing level [12,14]. The Garner task is often used to test whether different dimensions of a task can be processed independently and therefore has to be combined with another paradigm [15–18]. The Garner effect seems to evolve during filtering of the irrelevant dimension in early processing stages [15–17,19]. Therefore, the Garner effect is an obvious manipulation for the investigation of visual filtering effects in the Eriksen flanker task by increasing the filtering needs in a combination of both Flanker- and Garner paradigms.

In the Eriksen flanker task identification of a central target stimulus is delayed when it is surrounded by two or more task-irrelevant stimulus features. For instance, subjects could be asked to indicate whether a central stimulus is blue or red while flanking distractor stimuli are either blue or red [7]. Thus pairings of target and distractor stimuli can be congruent or incongruent in color. In incongruent trials subjects have to ignore the irrelevant information coming from flanking distractor stimuli and preferentially process the target information. According to our hypothesis this depends on visual filtering mechanisms. The slowing in reaction times during incongruent in contrast to congruent Flanker trials is referred to as Flanker interference [20].

In the Garner speeded classification paradigm performance between blocks of different conditions is compared. In the baseline condition there is a variation of the relevant dimension (e.g. target stimuli) while the irrelevant dimension remains constant (e.g. distractor stimuli). In contrast, features of both dimensions vary unpredictably in the filtering condition. The slowing of reaction times due to the variation of the irrelevant dimension during filtering condition blocks is referred to as Garner interference [9,21–23].

Recently, Wendt and colleagues (2012) emphasized visual filtering mechanisms in a traditional Flanker task. In their first experiment, subjects had to respond to a central target stimulus and ignore flanker stimuli. They hypothesized that the flanker task would bias information processing towards the target-related stimulus location. To test this hypothesis the authors interspersed trials of a search task among blocks of the flanker task. In this search task, subjects had to identify a target digit, which might occur at a location associated with the target or the flankers. Subjects showed an advantage to detect probe digits presented at the central location compared to a control task without a flanker task. This finding demonstrates the role of visual filtering by narrowing the attentional focus to the central target position. Thus, both visual filtering mechanisms at an early level of information processing and inhibition processes at the stage of response selection probably contribute to the Flanker conflict resolution. On the neural level, Flanker interference seems to be resolved by recruitment of cognitive control and filter mechanisms through a fronto-parietal neuronal network including anterior cingulate cortex (ACC), middle frontal gyrus (MFG), inferior frontal gyrus (IFG), the inferior (IPL) as well as the superior parietal lobule (SPL) [7,24].

There were several studies using event related potentials (ERP) to investigate the time course of Garner interference processing combined with a Stroop or a speech perception task. These studies indicate that additional attentional and filtering resources are needed to process the Garner effect at early perceptual stages [15–17,19]. This attentional effort seems to play an important role for filtering the unpredictable variation of distractors from the irrelevant dimension [10]. Therefore the main source of conflict in the Garner paradigm seems to be a stimulus-stimulus conflict where the random variation of the irrelevant dimension leads to an increase in filtering demands. Boenke and colleagues found a Garner effect on the P3 amplitude, which was larger in the baseline than the filtering condition [19]. Findings from a different study investigating filtering of distractors in a visual search task corroborate the reduced P3 amplitude in trials where visual filtering is needed compared to a condition with low filtering demands [25]. To the best of our knowledge, there is no study so far using functional magnetic resonance imaging (fMRI) to address the neural effects underlying the Garner effect and its modulation of a classical conflict task. However, neuroimaging studies on mechanisms of general spatial filtering of distractors, although not in terms of random variation as in the Garner paradigm, particularly suggest parietal regions such as the intraparietal sulcus (IPS) to play an important role in the filtering process [26–28].

Most of the studies mentioned above interpreted the slowing in reaction times in the Flanker task to be due to the distraction arising from irrelevant flankers that subjects have to filter out. Here, we used the Garner task to additionally increase filtering needs during the Flanker task by random and task irrelevant contextual variation in filtering blocks resulting from the Garner manipulation in a combined Flanker-Garner paradigm. Although visual filtering mechanisms seem to play a key role in the processing of both the Flanker and Garner paradigm, it is not clear whether they share or recruit different visual filtering mechanisms. In fact, filtering demands might differ between both paradigms. In the Garner paradigm visual filtering is necessary in filtering but not during the processing of baseline blocks. Furthermore, the Garner effect might be subject to short-term-memory processes because conflict effects in the Garner paradigm usually occur across blocks [17,19]. In the Flanker task, however, the importance of visual filtering changes from incongruent to congruent trials on a trial-by-trial basis. Therefore, a common filter mechanism would have to meet filtering demands in congruent and incongruent Flanker trials, but at the same time would have to be flexible to respond to increased filtering demands during filtering conditions of the Garner effect. These conditions could be also achieved by two separate and distinct filter mechanisms, as has been shown in previous studies on different conflict-specific cognitive control mechanisms [7,29].

In the present study we particularly addressed the question whether visual filter mechanisms during the processing of the Garner paradigm and the Flanker conflict results in the activation of different brain regions. We expected a fronto-parietal neuronal network to be involved in the resolution of the Flanker conflict and parietal brain regions to contribute to the filtering of random contextual variations in Garner filtering blocks.

## Materials and Methods

### Participants

Twenty-four healthy students from the Bremen University campus (Germany) participated in the experiment (11 male; mean age 23.3 years, *SD* = 2.25, age range 20–29 years). All subjects were right-handed according to the Edinburgh Handedness Inventory Scale [30] and had normal or corrected to normal vision. Subjects were screened for neurologic or psychiatric history and excluded from further examination in case of incidents reported during history taking. The study was conducted and designed in accordance with the Declaration of Helsinki [31], and all subjects gave informed and written consent for their participation in accordance with ethic and data security guidelines of the University of Bremen. The study was approved by the ethics committee of the University of Bremen.

### Stimulus material and trial sequence

**Fig 1:**
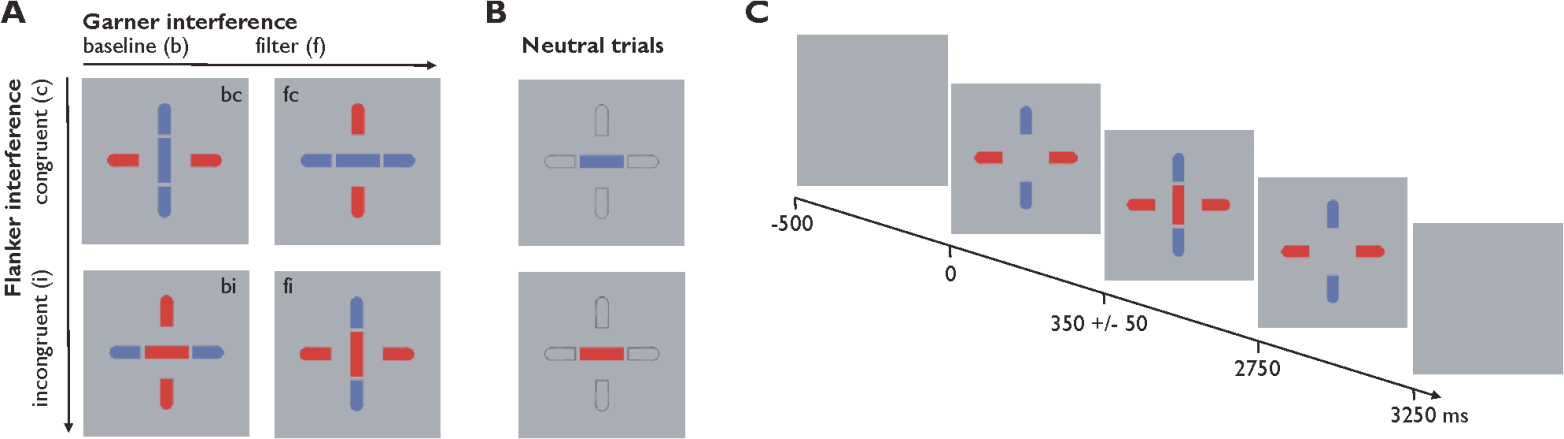
Stimuli and stimulus sequence. (A) Stimuli consisted of a central stimulus and four surrounding bars. The central stimulus could be either red or blue as well as in a vertical or horizontal orientation. The surrounding bars were displayed in a horizontal axis in blue paired with a vertical axis in red or vice versa. The central stimulus either matched the color of the surrounding bars in the same orientation (congruent trials) or not (incongruent trials). In baseline blocks the surrounding bars were held constant whereas they changed their color randomly during filtering blocks. (B) In neutral trials Flanker bars consisted of gray-framed stimuli to avoid interference during color discrimination of the central bar. (C) Stimulus sequence of an incongruent trial: After the short presentation of the Flanker bars a central bar appeared in the middle of the screen. Here, the red vertical central bar did not match the color of the vertical blue Flankers and therefore elicits a Flanker conflict.

Stimuli consisted of a central bar (relevant stimulus dimension) surrounded by four flankers (irrelevant stimulus dimension) (see Fig. 1A). The central bar could be either blue (CIELab color space, Lab 51, 4, -33) or red (Lab 50, 64, 45). The bar was presented either in a horizontal or a vertical orientation, which resulted in visual binding by forming a horizontal or a vertical line with the horizontal or vertical Flankers, respectively. The horizontal and vertical flankers were either blue or red (see Fig. 1A). Stimuli were presented within a grey square (Lab 71, 0, -2) on a black screen in the center of the screen.

Subjects were asked to indicate the color of the central bar with either their right or left index finger. Assignment of colors to the response buttons was counterbalanced across participants. Subjects were asked to respond as fast and accurate as possible.

During the task, subjects were confronted with two different conflict conditions. In congruent Flanker trials the color of the central bar matched the color of the surrounding flankers according to the spatial orientation of the bar, thus building a monochromatic line and leading to a more simple task condition. In incongruent Flanker trials, where the color of the central bar did not match the color of the surrounding flankers, there was a dichromatic line eliciting the Flanker conflict. Here, subjects had to filter out the information coming from the surrounding flankers. Each trial consisted of congruent and incongruent elements, but the Flanker conflict was only induced through visual binding of the central stimulus and the surrounding bars along the spatial orientation of the central bar.

With respect to the Garner effect we manipulated the level of visual filtering by randomly varying the irrelevant stimulus dimension. While the configuration of the surrounding flankers remained static during Garner baseline blocks (i.e., during the whole block one axis was red whereas the other one was blue), they changed their color randomly in the Garner filtering condition on a trial-by-trial basis (i.e., in every trial either the vertical or the horizontal axis was blue while the opposite one was red). Thus, subjects could rely on context stability in baseline blocks, whereas they had to filter out the irrelevant contextual variation during filtering blocks. Additionally, the experimental design also included a neutral baseline condition. During neutral trials the surrounding Flanker bars were grey (CIELab color space, Lab 71, 0, -2) thereby ensuring that there was no conflicting color information introduced by the Flankers (see Fig. 1B).

The Garner baseline condition consisted of 96 trials, where the Flanker dimension was congruent (baseline congruent (bc)), and of 96 trials, where the flanker dimension was incongruent (baseline incongruent (bi)). The Garner filtering condition consisted of 96 congruent Flanker trials (filtering congruent (fc)) and 96 incongruent Flanker trials (filtering incongruent (fi)). In addition there was a total of 192 neutral trials.

Each trial started with the presentation of the surrounding bars for 300 ± 50 ms followed by the presentation of the target stimulus for 300 ms. Thereafter, the surrounding bars were displayed again for another 2150 ms (see Fig. 1C). We decided to extend the post-target presentation time of the surrounding bars to ensure that participants were aware of the change of the color axes between the different Garner trials. Between stimuli there was a blank screen with a grey square displayed for 500 ms.

Stimuli were presented in randomized order and in blocks of 24 trials separated by short breaks (6000 ms). Each block consisted of an equal number of congruent and incongruent Flanker trials. This mixed block/event-related design allowed us to separate the underlying mechanisms of within-trial Flanker and between-trial Garner interference [32,33]. Moreover, the design meets the demands of a factorial task-crossing design, which allows for a factorial analysis of conflicts without task switching effects [6]. Since trials show different activations depending on the preceding trial, we balanced the trial sequences for each block in order to avoid sequential congruency effects, such as the Gratton effect of post-conflict adjustment [34]. Hence, each trial type had the same probability to be preceded by any other trial type. The sequence of blocks was counterbalanced between subjects. The experiment consisted of two parts that were separated by a 1 minute break. Although the whole experiment was measured within one scanning session, we refer to the first and second part as run 1 and 2, respectively. Each run included twelve blocks. Every run started and finished with two neutral blocks. In between there were eight conflict blocks with alternating baseline and filtering blocks. The different runs started either with a baseline or a filtering block to avoid order effects. Stimuli were projected via a JVC video projector onto a projection screen positioned at the rear end of the fMRI scanner with a viewing distance of about 38 cm using Presentation–Software (Neurobehavioral Systems; https://nbs.neuro-bs.com).

We conducted a behavioral pilot study to test the validity of our experimental design. This pilot study included 16 subjects (mean age 22 years (*SD* = 2), 13 female) and demonstrated reliable Flanker and Garner effects in reaction times (RT, Flanker (*F*_*1*,*15*_ = 5.511, *p* < 0.05), Garner (*F*_*1*,*15*_ = 19.01, *p* < 0.001)) indicated by a repeated measures ANOVA including the within-subject factors Flanker (congruent, incongruent), and Garner (baseline, filter). Additionally, a detailed analysis of behavioral pilot data also showed a decrease of RTs over the course of the whole experiment. We therefore performed a runwise analysis comparing the data separately for the first and the second experimental run and found a significant order effect with overall reaction times decreasing significantly from the first to the second run (*F*_*1*,*15*_ = 8.511, *p* < 0.05). We assumed this finding to indicate task-related learning.

### Behavioral data analysis

Conflict effects were analyzed both for the whole experiment and for the first and second experimental run separately to investigate conflict effects over time. First, behavioral data were subjected to a 2 x 2 repeated measurement ANOVA with the within-subject factors *Garner* (baseline, filtering) and *Flanker* (congruent, incongruent). As Garner interference occurs between trials, Garner effects are likely to involve memory processes [17,19]. Furthermore, as data from our pilot study suggested a task-related learning process we performed a second analysis to investigate conflict effects across the two experimental runs. Behavioral data were subjected to a 2 x 2 x 2 repeated measurement ANOVA with the within-subject factors *experimental run* (1^st^ and 2^nd^), *Garner* (baseline, filtering) and *Flanker* (congruent, incongruent). As there was no significant main effect for the factor Garner on the level of the whole experiment, we further investigated the variability of the Garner conflict by analyzing Garner interference in Flanker congruent (fc—bc) and incongruent trials (fi—bi) separately across the two experimental runs. Conflict indices were subjected to a 2 x 2 repeated measures ANOVA with the within-subject factors Garner (Flanker congruent, Flanker incongruent) and experimental run (first and second). We included only trials with a correct response and excluded trials with reaction times less than 100 ms or more than three standard deviations of the mean reaction time. Behavioral measures were analyzed by the statistical software package PASW 18.

### Image acquisition

Imaging data were obtained on a SIEMENS Magnetom Allegra System (Siemens, Erlangen, Germany) using a T2*-weighted gradient echo planar imaging (EPI) sequence (28 contiguous axial slices aligned to the AC-PC plane, slice thickness 4 mm, no gap, TR = 1.5 s, TE = 30 ms, FA = 73°, in-plane resolution 3 x 3 mm), and using a manufacturer supplied circularly polarized head coil to measure changes in blood oxygenation level-dependent (BOLD) signals. We additionally obtained high resolution magnetization prepared rapid acquisition gradient echo (MPRAGE) T1-weighted sequence (176 contiguous slices, TR = 2.3 s, TE = 4.38 ms, TI = 900 ms, FA = 8°, FOV 296 x 296 mm, in-plane resolution 1 x 1 mm, slice thickness 1 mm) in sagittal orientation to get anatomical images from each subject.

### Image analysis

For preprocessing and statistical analysis we used the Statistical Parametric Mapping software (SPM, Version 8; Wellcome Department of Imaging Neuroscience, London, UK). First, functional images were realigned to the mean image of each data set after motion estimation. Segmentation of anatomical images revealed warping parameters to normalize functional images, which were co-registered to the anatomical image beforehand. During normalization functional images were resampled to 2 x 2 x 2.66 mm voxel size. Normalized images were spatially smoothed using a non-isotropic Gaussian kernel of FWHM 8 x 8 x 10.64 mm with the purpose of decreasing differences in individual structural brain anatomy and increasing the signal-to-noise ratio (SNR). Images were high-pass filtered (128 s) to remove low-frequency signal drifts. We used a first-order autoregressive model (AR-1) for estimating temporal autocorrelations by using restricted maximum likelihood estimates of variance components.

To model the functional data, delta functions defined by the onset of a stimulus on a trial-by-trial basis were convolved with a hemodynamic response function (HRF) and its first temporal derivative. First and second level data were analyzed using a mixed-effect general linear model (GLM) approach [35]. All experimental conditions were entered into the GLM as separate regressors for the following trials with correct responses: neutral, baseline congruent, baseline incongruent, filtering congruent and filtering incongruent. An additional regressor for all error trials as well as a regressor for the remaining part consisting of instructions and breaks was entered into the GLM. Furthermore, six motion correction parameters were added as regressors of no interest to minimize false positive activations due to task correlated motion [36]. On the single subject level, contrasts were created by comparing filtering and baseline trials [filtering (fc and fi) > baseline (bc and bi)], as well as incongruent and congruent trials [incongruent (bi and fi) > congruent (bc and fc)]. We expected enhanced filtering demands in trials with a combined Garner and Flanker conflict. Therefore, regions of distinct activation for double conflict trials (incongruent trials in filtering blocks (fi)) were analyzed by an interaction analysis, which compared activations during filter incongruent (fi) trials with activations during the remaining trials (fc, bi and bc; [(fi > fc) > (bi > bc)]). Functional data analyses followed the same logic as the analysis of behavioral effects. We investigated the main effect for the factor Garner (filtering vs. baseline) and Flanker (incongruent vs. congruent) as well as the interaction contrast for the whole experiment as well as for the first and second experimental run separately.

**Fig 2:**
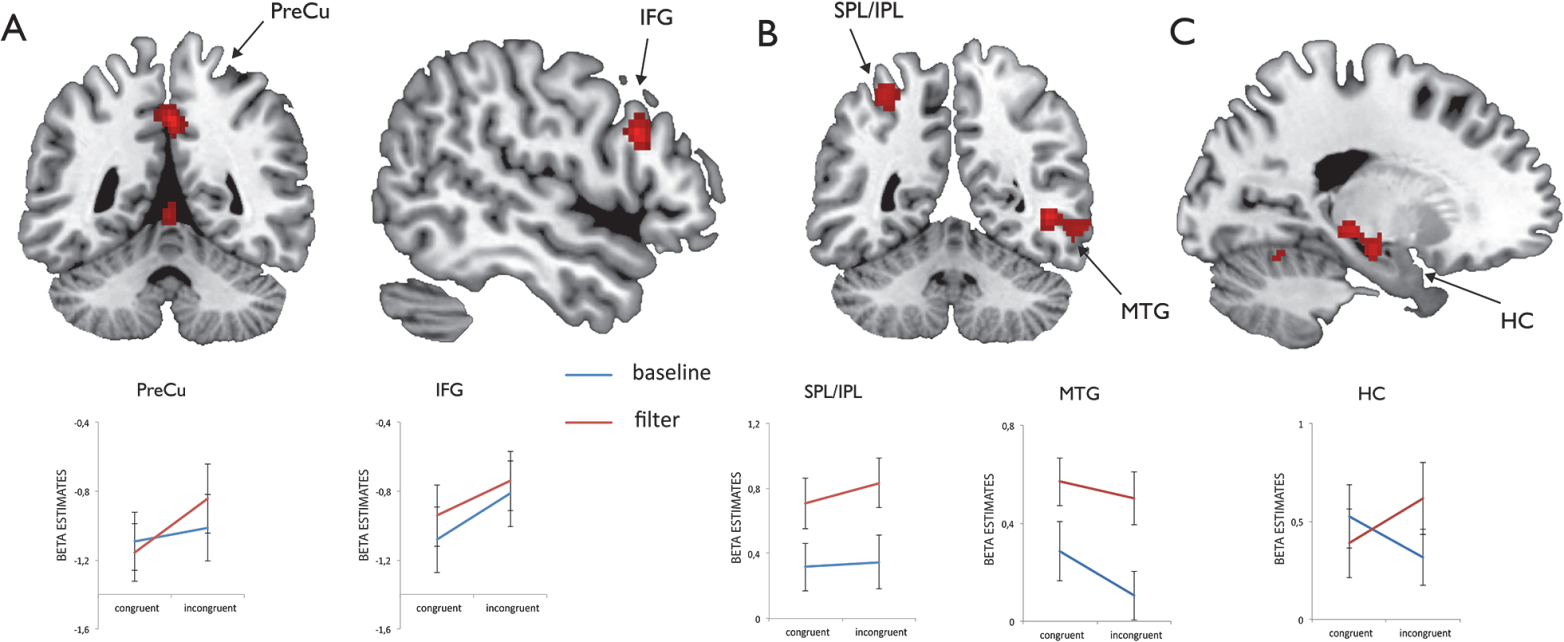
FMRI data for the whole experiment. Functional activations for the Flanker (A), the Garner (B) and the interaction contrast (C) across the whole experiment as well as mean beta estimates extracted from a sphere with 4 mm radius around peak locations. The interaction contrast compared activations during incongruent trials in Garner filtering blocks (fi) with activations during the remaining trials (fc, bi and bc). Abbreviations: *HC* hippocampus, *IFG* inferior frontal gyrus, *MTG* middle temporal gyrus, *IPL* inferior parietal lobule, *PreCu* precuneus, *SPL* superior parietal lobule.

**Fig 3:**
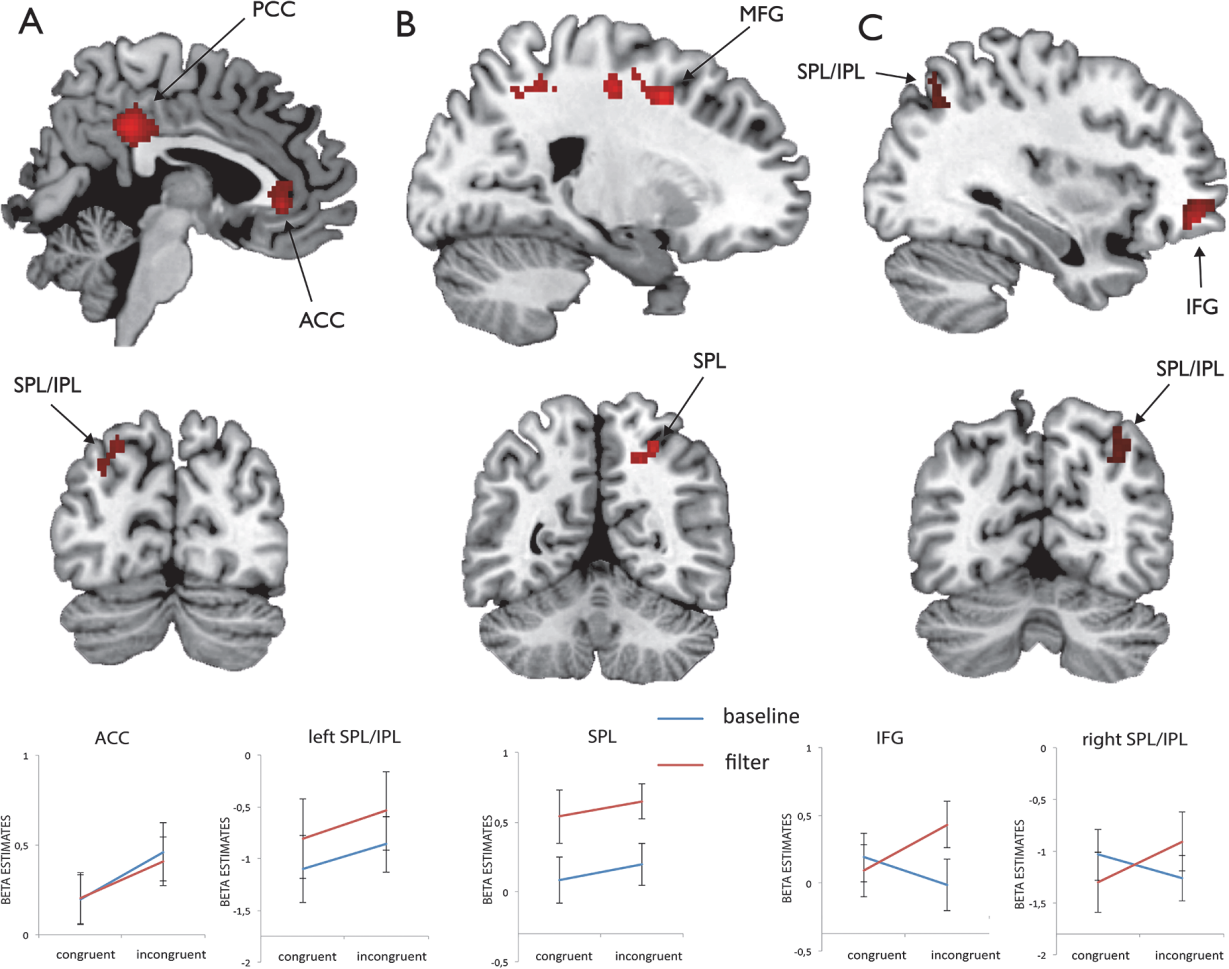
FMRI data for the first experimental run. Functional activations for the Flanker (A), the Garner (B) and the interaction contrast (C) in the first experimental run as well as mean beta estimates extracted from a sphere with 4 mm radius around peak locations. The interaction contrast compared activations during incongruent trials in Garner filtering blocks (fi) with activations during the remaining trials (fc, bi and bc). Abbreviations: *ACC* anterior cingulate cortex, *PCC* posterior cingulate cortex, *MFG* middle frontal gyrus, *SPL* superior parietal lobule, *IPL* inferior parietal lobule, *IFG* inferior frontal gyrus.

**Fig 4:**
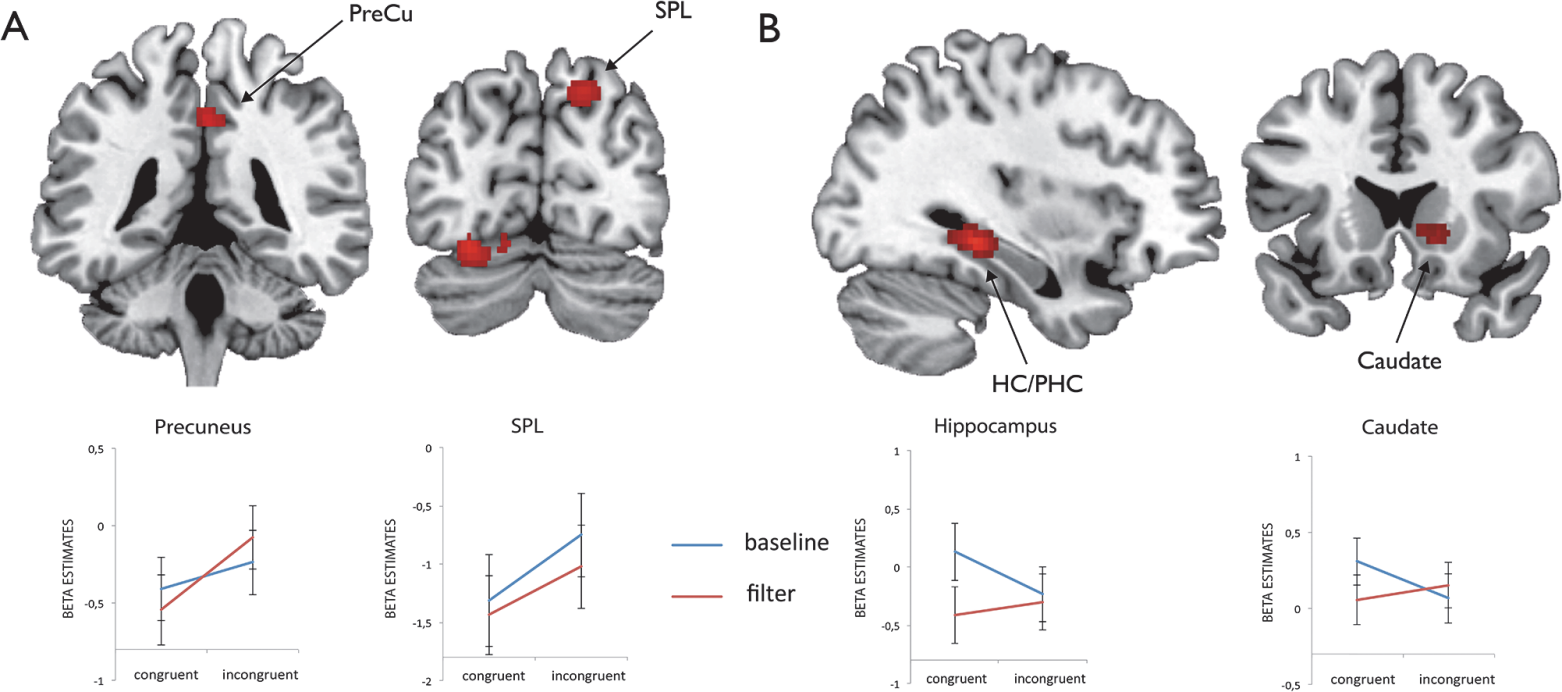
FMRI data for the second experimental run. Activations for the Flanker (A) and the interaction contrast (B) derived from the second experimental run as well as mean beta estimates extracted from a sphere with 4 mm radius around peak locations. Abbreviations: *SPL* superior parietal lobe, *PreCu* precuneus, *HC* hippocampus, *PHC* parahippocampal cortex.

First level contrasts were subjected to a second level one-sample t-test. Activations were thresholded at a combined voxel and cluster-size threshold of *p*< 0.005 (uncorrected) and a cluster extent of *k* = 50 following the procedure introduced in earlier studies on the topic [37]. For several peak activations (as derived from the contrasts as displayed in Figs. 2, 3 and 4) we extracted mean beta estimates in a sphere with 4 mm radius around peak locations for illustrative purposes.

## Results

### Behavioral data

#### Whole experiment

With respect to reaction times (RT; see Table 1) we found a significant main effect for the factor Flanker (F_1,23_ = 10.206, p < 0.005), but not for the factor Garner (F_1,23_ = 0.488, p = 0.492). Reaction times in incongruent (M = 487 ms, SEM = 13) compared to congruent Flanker trials (M = 476 ms, SEM = 11) were significantly increased. There was no significant Flanker x Garner interaction (F_1,23_ = 0.897, p = 0.354). We did not analyze error rates because the overall performance accuracy was above 98 percent.

**Table 1:** Reaction times (RT; ms) for the different experimental trials: Results are reported for (a) the whole experiment as well as (b) the first and (c) second experimental run separately.

Experimental trials					
	n	bc	bi	fc	fi
*(a) Whole experiment*					
RT	444 (9)	476 (11)	489 (14)	476 (10)	485 (12)
*(b) First run*					
RT	448 (10)	481 (11)	488 (12)	481 (11)	494 (14)
*(c) Second run*					
RT	441 (9)	467 (11)	490 (16)	471 (11)	476 (11)

neutral (n), baseline congruent (bc), baseline incongruent (bi), filtering congruent (fc) and filtering incongruent (fi). Numbers in brackets show the standard error of the mean (SEM).

#### Runwise analysis

A repeated measures 2 x 2 x 2 ANOVA for reaction times with the within-subject factors experimental block (first, second), Garner (baseline, filtering) and Flanker (congruent, incongruent) was used to investigate conflict effects and conflict adaptation over time. This ANOVA showed a significant main effect for the factor Flanker (F_1,23_ = 9.135, p < 0.01), a non-significant effect for the factor experimental run (F_1,23_ = 4.105, p = 0.055) and a significant Garner x Flanker x experimental run interaction (F_1,23_ = 4.832, p < 0.05). RTs in incongruent (M = 486 ms, SEM = 13) compared to congruent trials (M = 476 ms, SEM = 11) were significantly increased. RTs in the second run (M = 476 ms, SEM = 12) were lower than in the first run (M = 486 ms, SEM = 12). The significant Garner x Flanker x experimental run interaction is caused by a shift of the Flanker effect from the Garner filtering (Run 1: filtering congruent: M = 481, SEM = 11, filtering incongruent: M = 494, SEM = 14) to the Garner baseline condition (Run 2: baseline congruent: M = 467, SEM = 11, filtering incongruent: M = 490, SEM = 16). Paired t-tests comparing RT differences between the Flanker congruent and incongruent condition within the baseline and the filtering condition of the garner in each experimental run confirmed the change of the Flanker interference effects (Run 1: Garner baseline: Flanker, t_23_ = -1.322, p = 0.199; Garner filtering: Flanker, t_23_ = -2.093, p < 0.05; Run 2: Garner baseline: Flanker, t_23_ = -3.133, p < 0.05; Garner filtering: Flanker, t_23_ = -1.817, p = 0.082). There was no significant main effect for the factor Garner (F_1,23_ = 0.147, p = 0.705) nor for the experimental run x Garner (F_1,23_ = 2.651, p = 0.117), experimental run x Flanker (F_1,23_ = 1.316, p = 0.263) or Flanker x Garner interaction (F_1,23_ = 1,279, p = 0.270). A repeated measures ANOVA addressing the variability of Garner interference across experimental runs showed no significant main effect for the factor Garner (F_1,23_ = 1.279, p = 0.27) or experimental run (F_1,23_ = 2.651, p = 0.117), but a significant Garner x experimental run interaction in RTs (F_1,23_ = 4.832, p < 0.05). This interaction effect is due to higher Garner interference in incongruent Flanker trials (fi—bi) in the first compared to the second experimental block (Block 1: M_fi-bi_ = 7, SEM = 6; Block 2: M_fi-bi_ = -14, SEM = 7). Paired t-tests comparing conflict effects in both experimental runs confirmed the decrease in Garner interference in the second compared to the first experimental run specifically in Flanker incongruent trials (fi-bi) (Garner interference in Flanker congruent trials (fc-bc) across runs, t_23_ = -0.666, p = 0.512; Garner interference in Flanker incongruent trials (fi-bi) across runs, t_23_ = 2.337, p < 0.05).

### Imaging data

#### Whole experiment

The whole brain analysis revealed a significant increase in BOLD activity for the main effect of the factor Flanker ((fi + bi) > (fc + bc)) in the right inferior frontal gyrus, the left superior frontal gyrus, the left precuneus (PreCu) extending to the right precuneus, the right caudate extending to the anterior cingulate cortex and the left and right cerebellum extending to the thalamus (see Fig. 2A and Table 2). There was a significant activation for the main effect of the factor Garner ((fc + fi) > (bc + bi)) in the left superior and inferior parietal lobule. Furthermore we found significant activation in the right middle temporal gyrus (MTG) and the left lingual gyrus (Fig. 2B). Interaction analysis revealed regions of specific activation for incongruent trials in Garner filtering blocks involving the right middle temporal gyrus, the left hippocampus extending to the left substantia nigra, the left fusiform gyrus and the bilateral cerebellum (Fig. 2C). In a second step we applied a runwise analysis for investigating both Flanker and Garner main effects in each experimental run separately.

**Table 2:** Peak activations derived from an analysis of the Flanker [bi fi > bc fc], the Garner conflict [fc fi > bc bi] and an interaction analysis [(fi > fc) > (bi > bc)] across the whole experiment.

	Region	MNI coordinates	*t*	Cluster size
*FLANKER*				
	L superior frontal gyrus	-4 32 54	4.35	54
	R inferior frontal gyrus	50 18 27	5.37	87
	L precuneus	0 -48 43	5.47	95
	R precuneus	2 -42 48	3.25	
	caudate	4 2 1	5.17	327
	caudate	-4 8 -2	4.25	
	anterior cingulate	6 20 -5	3.85	
	L cerebellum	0 -44 -2	4.75	359
	R cerebellum	12 -34 -5	4.13	
	L thalamus	-4 -22 9	4.11	
*GARNER*				
	R middle temporal gyrus	44 -50 -2	4.86	241
	R middle temporal gyrus	48 -60 -2	4.09	
	R middle temporal gyrus	64 -50 -7	4.28	
	L superior parietal lobule	-30 -58 54	3.95	204
	L inferior parietal lobule	-34 -50 54	3.86	
	L lingual gyrus	-30 -60 -5	4.04	89
	L lingual gyrus	-34 -72 -2	3.33	
*INTERACTION*				
	R middle temporal gyrus	48 0 -15	3.74	51
	L hippocampus	-18 -14 -15	4.35	176
	L substantia nigra	-14 -24 -13	4.22	
	L fusiform gyrus	-24 -58 -15	3.57	58
	R cerebellum	28 -44 -23	4.52	413
	R cerebellum	16 -40 -23	4.08	
	R cerebellum	30 -44 -31	3.80	
	L cerebellum	-32 -48 -26	3.81	63

#### Runwise analysis

In the first experimental run we found significant activation for the main effect of the factor Flanker in the left and right posterior and anterior cingulate cortex. Furthermore signal increases were found in the left superior and inferior parietal lobule (see Fig. 3A and Table 3). For the main effect of the factor Garner there was significant activation in the right middle frontal gyrus extending to the superior frontal gyrus, the right superior frontal gyrus extending to the medial frontal gyrus and the postcentral gyrus extending to the right superior parietal lobule (Fig. 3B). We used an interaction analysis to investigate activation that was specific for the double conflict condition (Flanker incongruent trials in Garner filtering blocks). This approach revealed signal increases in the right middle frontal gyrus, the right inferior frontal gyrus extending to the left medial frontal gyrus and the right IPL extending to SPL (Fig. 3C).

**Table 3:** Peak activations derived from an analysis of the Flanker [bi fi > bc fc] and the Garner conflict [fc fi > bc bi] and an interaction analysis [(fi > fc) > (bi > bc)] within the first experimental run.

	Region	MNI coordinates	*t*	Cluster size
*FLANKER*				
	R posterior cingulate	4 -38 35	6.04	311
	L posterior cingulate	-2 -24 35	3.10	
	L anterior cingulate	-4 26 -2	5.29	140
	R anterior cingulate	2 34 -2	4.61	
	R anterior cingulate	2 36 9	3.15	
	L superior parietal lobe	-22 -78 46	3.57	73
	L angular gyrus/IPL	-34 -70 32	3.36	
*GARNER*				
	R middle frontal gyrus	22 6 46	4.49	90
	R superior frontal gyrus	22 -6 54	2.96	
	R superior frontal gyrus	24 -16 48	3.95	72
	R medial frontal gyrus	16 -18 56	3.19	
	R postcentral gyrus	26 -38 51	3.71	151
	R superior parietal lobe	26 -56 51	3.61	
	R superior parietal lobe	18 -54 46	3.46	
*INTERACTION*				
	R middle frontal gyrus	42 52 -10	7.77	202
	R inferior frontal gyrus	28 16 -13	4.60	112
	L medial frontal gyrus	-10 32 38	4.27	86
	L medial frontal gyrus	-8 40 35	3.95	
	R inferior parietal lobule	34 -66 43	3.38	76
	R superior parietal lobule	32 -66 51	3.24	
	R inferior parietal lobule	40 -50 38	3.05	

**Fig 5:**
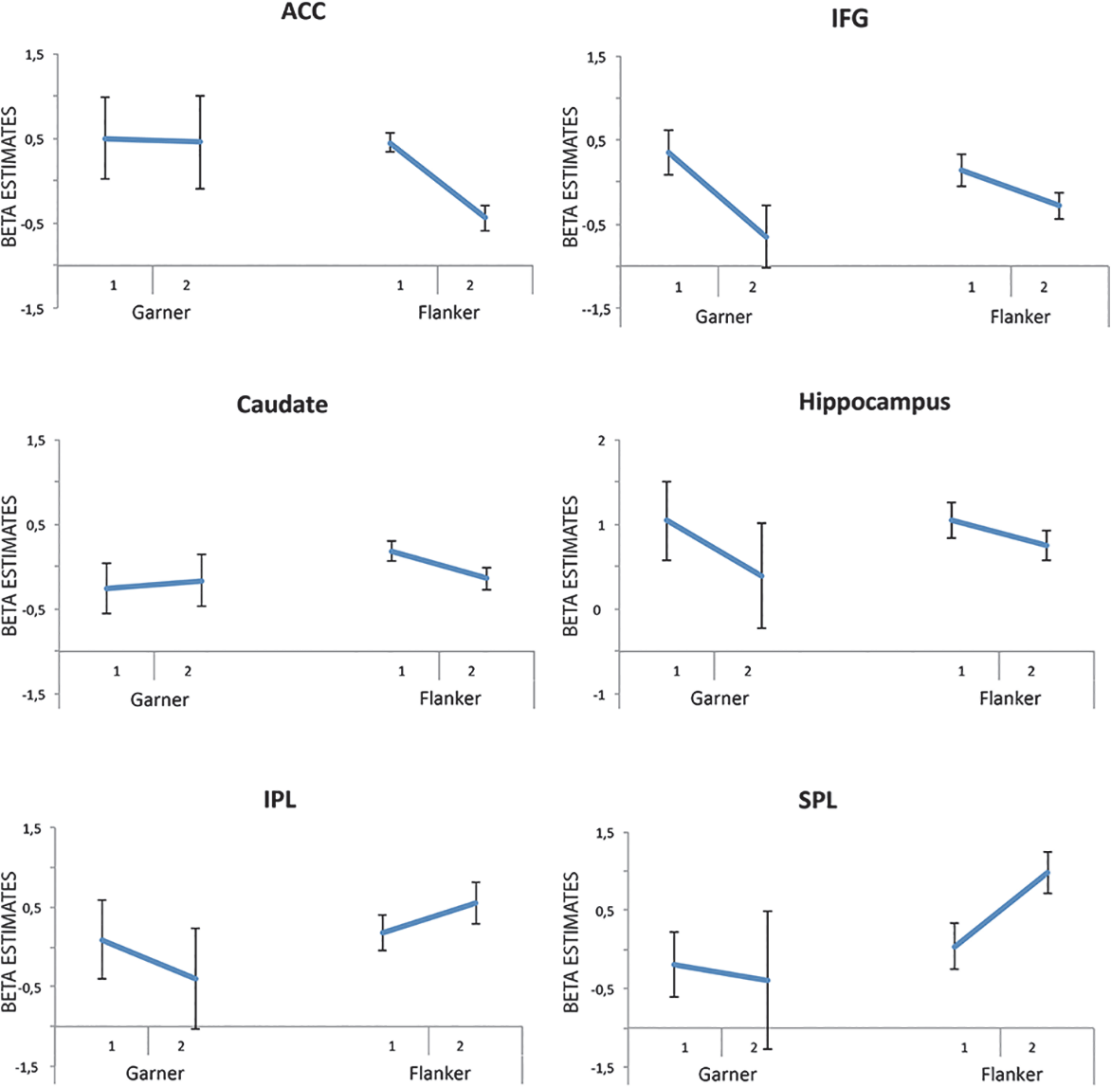
Beta estimates for the Flanker and the Garner contrast across the first and second experimental run. Abbreviations: *IPL* inferior parietal lobe, *SPL* superior parietal lobe, *IFG* inferior frontal gyrus, *ACC* anterior cingulate cortex, *1* and *2* indicate first and second experimental runs.

In the second experimental run the whole brain analysis for Flanker incongruent in comparison to Flanker congruent trials revealed stronger activations in the right precuneus and the right superior parietal lobule and also in the right inferior temporal gyrus and the left occipital gyrus extending to the cerebellum (Fig. 4A and Table 4). The main effect of the factor Garner showed no significant results. Interaction analyses revealed activation in the right hippocampus extending to the parahippocampal cortex, the left superior temporal gyrus and the right caudate that was specific to incongruent trials in Garner filtering blocks (Fig. 4B). We extracted mean beta estimates for peak activations in the ACC, IFG, the hippocampus, the caudate as well as IPL and SPL and calculated conflict effects for the first and second experimental run (filter—baseline blocks; incongruent—congruent trials) in order to document the development of conflict effects across experimental runs (see Fig. 5).

**Table 4:** Peak activations derived from an analysis of the Flanker conflict [bi fi > bc fc] and an interaction analysis [(fi > fc) > (bi > bc)] across the second experimental run.

	Region	MNI coordinates	*t*	Cluster size
*FLANKER*				
	R inferior temporal gyrus	32 -60 -15	4.53	93
	L occipital gyrus	-28 -78 -15	4.06	144
	L occipital gyrus	-12 -76 -15	3.30	
	L cerebellum	-14 -84 -15	3.28	
	R precuneus	2 -42 43	3.78	77
	R SPL/precuneus	18 -78 46	3.93	75
*INTERACTION*				
	R hippocampus	34 -34 -7	6.02	182
	R parahippocampal cortex	-28 -38 -10	3.62	
	R parahippocampal cortex	28 -50 -10	3.38	
	L superior temporal gyrus	-58 -28 -1	4.21	112
	L superior temporal gyrus	-66 -30 3	3.25	
	R caudate head	16 18 -2	3.64	60

## Discussion

The present study aimed at investigating the neuronal mechanisms underlying Flanker and Garner interference resolution in a combined Garner-Flanker task by means of fMRI. In particular, we were interested in whether both behavioral and imaging data point to different filter mechanisms involved in the various types of conflict resolution. The behavioral analysis revealed a significant Flanker effect, but no significant effect of the Garner conflict. Based on the findings of a previous behavioral pilot study pointing to implicit learning processes within the whole experimental design and the fact that Garner interference occurs between trials [17,19], we performed an additional data analysis to investigate conflict adaptation due to learning processes over time. This analysis again showed significant differences between the first and the second run of the experiment. The Flanker conflict main effect remained constant, but was shifted from the Garner filtering to the baseline condition. Garner interference in Flanker incongruent trials was significantly decreased in the second experimental run. This was accompanied by decreased overall reaction times in the second compared to the first experimental run.

Both conflict effects were associated with a fronto-parietal activation pattern. The pattern related to Flanker interference resolution comprised the anterior cingulate cortex, inferior frontal gyrus, the precuneus and the superior and inferior parietal lobule, a finding which was also reported by other authors investigating the neuronal correlates of Flanker conflict processing [24]. On the other hand, filtering of irrelevant and unpredictable variation of context as induced by the Garner paradigm resulted in an activation pattern comprising middle frontal gyrus, middle temporal gyrus, lingual gyrus, SPL and IPL.

### Visual filtering

Flanker related activation in the IFG, the precuneus as well as in IPL and SPL has also been shown in several Flanker conflict studies [7,24,38–40] thus corroborating the view that prefrontal areas are involved in the adjustment of control by modulating parietal areas in a top-down fashion. Previous neuroimaging studies demonstrated parietal regions (intra-parietal sulcus, the inferior and superior parietal lobule) to be engaged in filtering out irrelevant visual information [26–28]. Friedman-Hill and colleagues (2003) reported deficits in an orientation and face discrimination task in a patient with bilateral lesions in the parietal cortex. While the patient did not show problems with either task in general, he presented severe deficits with both tasks in the presence of distractors. The authors concluded that regions in the posterior parietal cortex (PPC) are crucial for the filtering of distractors, a finding which is also supported by other studies [26,27]. Although our findings are in line with studies investigating visual filtering, it is not possible to rule out effects from a response-based conflict that might arise at the level of response preparation. Nevertheless, we did not find activation in regions such as the premotor area as should be expected for response-based conflicts [41]. Therefore, parietal activation in the Flanker task might reflect visual filtering mechanisms that seem to play an important role during Flanker conflict resolution by narrowing the attentional focus to the target position [11].

Activation in the MFG, MTG, lingual gyrus, IPL and SPL related to the Garner effect so far is only supported by data from event-related potentials studies. Several studies suggest that the human brain engages attentional resources during stimulus perception in Garner filtering relative to baseline blocks [10,15–17,19]. In the Garner paradigm this attentional effort might be required to extract information from the task-relevant dimension while filtering out the unpredictable variation of the irrelevant one. Activation in IPL and SPL related to the Garner effect supports findings from Boenke and colleagues (2009). They found a Garner effect in the amplitude of the P3, i.e. the amplitude was lower in filtering than in baseline trials. Additionally, the maximum of the P3 amplitude in all Garner conditions was found at Cz. A study from Akyürek and Schubö (2003) found the same effect in the amplitude of the P3 in a study investigating the filtering of distractors in a visual search task. In filtering conditions subjects had to ignore and filter out lines that were tilted in the wrong direction. The amplitude of the P3 was lower in trials with enhanced filtering demands. In an earlier study from the same group fMRI was used in almost the same paradigm to investigate the filtering of distractors [42]. The authors reported in addition to the precuneus and SPL also the MTG and middle occipital gyrus (MOG) to be associated with the filtering of irrelevant distractors. Therefore, it is likely that our present findings might reflect visual filtering mechanisms that are necessary to ignore random contextual variation in Garner filtering blocks.

However, filtering demands differ between both types of tasks. For resolving the Flanker conflict, the subject has to act on a trial-by-trial basis as conflict only occurs in incongruent but not in congruent trials. Therefore, additional attentional resources have to be engaged during incongruent trials in order to focus on the central stimulus and to ignore irrelevant flankers simultaneously. In contrast, the Garner effect arises across trials and blocks due to the unpredictable variation of context, which exists in filtering blocks but not in baseline blocks. Therefore, the cognitive system has to recruit additional cognitive resources during filtering blocks. Our present data indicate that there are similar neural mechanisms involved in both filtering information of irrelevant distractors on a trial-by-trial basis in the Flanker conflict and in sustained filtering of unpredictable and irrelevant context variation during filtering blocks in the Garner conflict.

### Variability of conflicts effects across experimental blocks

If both conflict effects would rely on similar visual filtering mechanisms one would expect even enhanced filtering demands in Flanker incongruent trials during Garner filtering blocks (i.e. fi trials). This assumption should lead to increased RTs in incongruent trials in Garner filtering blocks and probably to an enhanced recruitment of neural resources. The behavioral data of the first experimental run suggest the need of filtering in the Flanker task and even enhanced filtering demands in incongruent trials during filtering blocks (fi) where both conflicts were present in the same trial. Interaction analyses corroborated this view. While the separate resolution of both conflicts recruited frontal and parietal brain regions (ACC, IFG, SFG, MFG, PreCu, IPL, SPL), the resolution of both conflicts simultaneously resulted in additional activation in distinct regions (MFG, IFG, MeFG, IPL, SPL).

However, we found substantial differences in the second compared to the first experimental run. First, overall RTs were decreased compared to the first run. Second, while there was a Flanker effect in Garner filtering blocks in the first experimental run, we found a significant Flanker effect only in the Garner baseline condition in the second experimental run. Third, Garner interference was reduced in Flanker incongruent trials in the second compared to the first run. Interaction analyses revealed distinct activation for incongruent trials in Garner filtering blocks in the hippocampus, the parahippocampal cortex, the superior temporal gyrus and the caudate nucleus. Brain areas in the medial temporal lobe (MTL) as well as the basal ganglia are involved in various kinds of learning [43,44]. Whereas MTL structures seem to be important for developing specific stimulus representations and flexible relational rules, the basal ganglia, especially the caudate nucleus, are important for stimulus-response associations [45,46]. The caudate nucleus also seems to be involved in shifting the decision criterion on a trial-by-trial basis in situations where the conditions for decisions change [47]. In the present combined Flanker and Garner task it is likely that subjects learned that filtering blocks are associated with increased task-difficulty in the first experimental run leading to perceptual learning mechanisms such as attentional weighting, which results in increased attention to relevant or decreased attention to irrelevant features [48]. In the second experimental run subjects showed improved performance in incongruent trials in filtering blocks possibly relying on stimulus representations and stimulus-response associations in the hippocampus, parahippocampal cortex and the caudate nucleus [45,46]. This would explain the missing Flanker effect in Garner filtering blocks. The decrease in beta values in frontal regions like IFG and ACC, which was accompanied by an increase in beta values in parietal regions like IPL and SPL in the second compared to the first experimental run might be associated with this kind of task-related learning (see Fig. 5). Additional evidence for task-related learning procedures is the decreased reaction time in the second compared to the first experimental run, which was more pronounced in Garner filtering blocks. The finding that subjects specifically increased performance in the Garner filtering blocks is corroborated by a study from Dosher and colleagues (2010). In this study the authors were interested in perceptual learning and shared attention between objects. They found that the deficit related to shared attention can be reduced through perceptual learning. Particularly, they showed the strongest learning effects in the condition, which was most challenging in terms of attention capturing [49].

In general Garner interference in behavioral data was limited to the Flanker incongruent condition and was rather weak. These weak effects differed from previous studies using Garner paradigms in the auditory domain, where Garner interference in the range from 43 to 159 ms could be observed [10,16,17]. However, in contrast to the present approach, these studies used highly integral auditory stimuli like timbre dimensions, vowels, and talker identity. For the visual domain Boenke and colleagues (2009) used a variation of either global or local stimulus features to elicit Garner interference and observed a Garner interference of 12 ms magnitude. In contrast to the study by Boenke and colleagues (2009), variation of the irrelevant dimension in the present experiment is rather subtle and therefore might account for the weaker Garner interference effects of 7–14 ms magnitude. Furthermore, we suppose that learning related effects across experimental blocks, as outlined above, might be responsible for the lack of significant Garner interference on whole experiment level.

## Conclusion

Taken together, our results suggest that filtering needs in the Flanker conflict task can be modulated using the Garner paradigm. Similar control mechanisms seem to be involved in Garner and Flanker conflict processing in the combined task used in the present study. The resolution of both effects recruits similar parietal brain regions such as IPL and SPL, which indicate the involvement of visual filtering processes. Furthermore, interaction effects in behavioral as well as fMRI data suggest that both effects rely on similar processes and neuronal resources. However, our data also suggest variability of conflict effects across experimental runs, which might be due to task-related learning processes. The interaction between both effects as well as the variability of conflict effects over time needs further investigation.
